# Bighorn sheep show similar in‐host responses to the same pathogen strain in two contrasting environments

**DOI:** 10.1002/ece3.9109

**Published:** 2022-07-17

**Authors:** Kezia R. Manlove, Annette Roug, Kylie Sinclair, Lauren E. Ricci, Kent R. Hersey, Cameron Martinez, Michael A. Martinez, Kerry Mower, Talisa Ortega, Eric Rominger, Caitlin Ruhl, Nicole Tatman, Jace Taylor

**Affiliations:** ^1^ Department of Wildland Resources and Ecology Center Utah State University Logan Utah USA; ^2^ Utah Division of Wildlife Resources Salt Lake City Utah USA; ^3^ Centre for Veterinary Wildlife Research, Faculty of Veterinary Science University of Pretoria Onderstepoort South Africa; ^4^ Taos Pueblo Division of Natural Resources Taos New Mexico USA; ^5^ New Mexico Department of Game and Fish Santa Fe New Mexico USA; ^6^ US Fish and Wildlife Service Washington District of Columbia USA

**Keywords:** bighorn sheep, ecoimmunology, *Mycoplasma ovipneumoniae*, wildlife disease, within‐host dynamics

## Abstract

Ecological context—the biotic and abiotic environment, along with its influence on population mixing dynamics and individual susceptibility—is thought to have major bearing on epidemic outcomes. However, direct comparisons of wildlife disease events in contrasting ecological contexts are often confounded by concurrent differences in host genetics, exposure histories, or pathogen strains. Here, we compare disease dynamics of a *Mycoplasma ovipneumoniae* spillover event that affected bighorn sheep populations in two contrasting ecological contexts. One event occurred on the herd's home range near the Rio Grande Gorge in New Mexico, while the other occurred in a captive facility at Hardware Ranch in Utah. While data collection regimens varied, general patterns of antibody signal strength and symptom emergence were conserved between the two sites. Symptoms appeared in the captive setting an average of 12.9 days postexposure, average time to seroconversion was 24.9 days, and clinical signs peaked at approximately 36 days postinfection. These patterns were consistent with serological testing and subsequent declines in symptom intensity in the free‐ranging herd. At the captive site, older animals exhibited more severe declines in body condition and loin thickness, higher symptom burdens, and slower antibody response to the pathogen than younger animals. Younger animals were more likely than older animals to clear infection by the time of sampling at both sites. The patterns presented here suggest that environment may not be a major determinant of epidemiological outcomes in the bighorn sheep—*M. ovipneumoniae* system, elevating the possibility that host‐ or pathogen‐factors may be responsible for observed variation.

## Graphical abstract

In this Nature Note, we compare disease dynamics of the same *Mycoplasma ovipneumoniae* strain in captive and free‐ranging bighorn sheep, and find strong parallels between dynamics in the two environments. The similarity in patterns across the two sites suggests that environmental factors may be less important that pathogen strain or host factors like genetics in producing variable outcomes among different bighorn sheep Mycoplasma ovipneumoniae disease events.

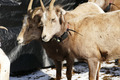

## 1 INTRODUCTION

Environmental context is thought to shape infectious disease transmission, yet directly comparing wildlife disease events in different environments is difficult due to concomitant differences in host genetics or pathogen strain. One pathogen for which ecological context may be important is *Mycoplasma ovipneumoniae*, a bacterial agent associated with infectious pneumonia in bighorn sheep (Besser et al., 2008, 2012, 2013). *M. ovipneumoniae* is an important player in the bighorn sheep respiratory disease complex (Dassanayake et al., 2010). Its introduction can lead to severe disease across all age classes of bighorn sheep, generating immediate‐term population declines of 10–90% (Cassirer et al., 2018). In the longer term, infected herds may experience years to decades of poor recruitment, driven by sustained infection among a few chronically infected adults (Cassirer et al., 2013; Cassirer & Sinclair, 2007; Garwood et al., 2020; Manlove et al., 2016; Plowright et al., 2017). Researchers have speculated that environmental context could have important bearing on the severity of *M. ovipneumoniae* spillover events (Butler et al., 2018; Manlove et al., 2014), and these speculations are supported by several comparative studies documenting variation in bighorn sheep disease severity across environments (e.g., Butler et al., 2018). There is evidence that herd mixing patterns may constrain disease burden in lambs (Manlove et al., 2014) and could therefore play a role in herd recovery (Almberg et al., 2021; Dugovich et al., 2017; Lula et al., 2020). Environmental context could also effect epidemiological patterns. For example, varying stress or nutrient availability could produce site‐to‐site variation in host susceptibility. However, direct comparisons of the same host genetics and pathogen strains in different environments are rare.

Here, we capitalize on intensive sampling surrounding a novel *M. ovipneumoniae* strain introduction event into members of a single bighorn sheep herd living in two different environments. One group of animals underwent the epidemic in captivity in a holding pen located at Hardware Ranch in northern Utah following capture and translocation from the wild. The other group experienced the epidemic while ranging freely on the herd's original home range near the Rio Grande Gorge in New Mexico. Although the environmental contexts differed, the events stemmed from the same *M. ovipneumoniae* strain, and animals in both groups had common genetics and health histories. This allowed us to compare disease and symptom progressions, antibody signal strengths, and epidemiological outcomes between the two contexts. Along with the comparisons, we present novel longitudinal data on disease progression during the epidemic, and estimate the incubation period, timing of peak of clinical signs, and patterns of antibody signal strength.

### 1.1 Study areas and disease event

The Taos Pueblo/Rio Grande Gorge in northern New Mexico (centered near 36.48°N, −105.73°W) is home to a reintroduced population of Rocky Mountain bighorn sheep (the RGG bighorn sheep population) that is jointly managed by the Taos Pueblo Tribe and New Mexico Department of Game and Fish (NMDGF). Most of the bighorn sheep habitat is within either Taos Pueblo tribal lands or Rio Grande del Norte National Monument, which is administered by the U.S. Bureau of Land Management. Elevation ranges from approximately 1800 to 2150 m (roughly 6000 to 7500 feet) above sea level. The population has been surveyed annually since 2008 using minimum counts implemented by a team of ground observers. The minimum count was 291 individuals in 2018 (NMDGF, 2019), and population size was estimated at 375–420 animals in the fall of 2019 (NMDGF unpublished data).

The RGG population was surveilled opportunistically for *M. ovipneumoniae* through serological and PCR testing based on blood and nasal swab samples, respectively. Sampling was conducted by NMDGF biologists and officers, or by hunter harvest samples gathered immediately upon kill. Hunters were instructed on proper sample collection and handling. The preponderance of surveillance samples were collected in 2018 (*n* = 48) and coincided with an impromptu population management ewe hunt. Only two hunter harvest samples were submitted in 2019. All samples gathered prior to the event reported here showed no serological or PCR evidence of *M. ovipneumoniae*. The herd was assumed to be *M. ovipneumoniae*‐free in the winter of 2020 because (1) the RGG herd had no known pneumonia history; (2) clinical signs had not been previously observed during survey events; and (3) biologist and hunter harvested samples submitted for diagnostic testing from 2018 until this event revealed no evidence of *M. ovipneumoniae*.

Based on these lines of evidence, Taos Pueblo Tribe and Utah Division of Wildlife Resources (UDWR) planned to translocate 24 animals from RGG to Antelope Island near Salt Lake City, UT on February 21st, 2020. The capture was conducted jointly by UDWR and the Taos Pueblo tribe. Female bighorn sheep were captured from across the RGG population by helicopter (Krausman et al., 1985). Animals were distributed across stalls in a sanitized UDWR livestock truck and driven overnight to Utah, arriving midday on February 22nd, 2020. Meanwhile, serum and nasal swab samples were flown directly from Taos, New Mexico to the Washington Animal Disease Diagnostic Laboratory (WADDL) immediately after capture, and PCR and competitive ELISA (cELISA) tests for *M. ovipneumoniae* (Ziegler et al., 2014) were conducted overnight. Although observers at the capture saw no clinical signs of pneumonia, even among animals running for sustained periods of time, PCR and cELISA test results indicated that six of the 24 translocated animals had current or previous *M. ovipneumoniae* infections.

Immediately upon receiving the test results, UDWR segregated the affected and unaffected animals. Affected animals were euthanized using approved UDWR protocols on February 23rd, and sampled a second time for *M. ovipneumoniae* postmortem. Gross examinations of lung tissue revealed minimal damage consistent with pneumonia among those animals. The 18 translocated animals that tested negative on both the PCR and the cELISA test were moved to a UDWR facility at Hardware Ranch (41.60°N, −111.56°W) on the evening of February 22nd, where they were chemically immobilized, moved to a 3 m high and 30 m diameter solid pen (approximately 700 m^2^ in area), and subsequently reversed. One animal (Eartag 32) died during chemical immobilization and two others (Eartags 33 and 34) died within 48 h of introduction to the pen, leaving a total of 15 animals in captivity for the preponderance of the study. Captive animals were fed a diet of grass hay, supplemented with a standard sheep pellet. Animals took several days to adapt to the new food but were regularly observed eating after the third day in the pen. Diarrhea was never observed in the captive animals. Captive animal research was conducted under Utah State University IACUC protocol #11117.

Following detection of *M. ovipneumoniae* among the translocated animals, NMDGF and the Taos Pueblo tribe initiated a parallel project sampling free‐ranging animals at the RGG herd. Animals were captured by ground darting using approved NMDGF protocols. Summer field investigation, fall surveys, and hunter harvest samples provided information on disease progression in the free‐ranging context.

## 2 METHODS

### 2.1 Data collection

#### 2.1.1 Animal handling and sampling

During the first capture and sampling event, animals were net‐gunned from a helicopter by a professional capture crew (Helicopter Wildlife Services, Austin, TX), hobbled and blindfolded, tranquilized with 14 mg haloperidol administered intramuscularly (Haloperidol, 20 mg/ml, Wildlife Pharmaceuticals, Laramie, WY), and transported to a processing location where trained personnel weighed and sampled the animals, applied global positioning system (GPS) collars (Advanced Telemetry Systems, Isanti, MN), administered 10 mg midazolam intravenously (Midazolam, 50 mg/ml, Wildlife Pharmaceuticals, Laramie, WY), and assessed body condition by ultrasound and palpation. The captive animals at Hardware Ranch were sampled three times each: on February 21st, 2020 prior to transportation to Utah; on March 12th, 2020; and at the time of euthanasia on March 26th, 2020 (both ante‐ and postmortem).

During the two captive resampling events, animals were herded into a corner of the pen and chemically immobilized by hand injection with 1.5 ml of butorphanol, azaperone, and medetomidine (BAM, Wildlife Pharmaceuticals, Laramie, WY). After injection, the bighorn sheep were released back into the pen. Once the animals were approachable, they were placed in sternal recumbency, blind folded, and sampled. All animals were reversed simultaneously after sampling with intramuscular injections of naltrexone and atipamezole (Kreeger & Arnemo, 2018). A UDWR veterinarian administered or supervised administration of all drugs.

Nasal and oropharyngeal swabs and serum were collected during the original capture and subsequent animal handling events at Hardware Ranch. Nasal swabs were collected by inserting a single DACRON™ swab into each nostril and gently swabbing the nasal mucosa by swirling the swab. One nasal swab was stored in Tryptic Soy Broth (TSB) for whole‐genome sequencing for a different project on the first and second captive sampling events. Diagnostic testing and strain typing were conducted by WADDL. *M. ovipneumoniae* DNA from six individuals were strain typed using a four‐locus MLST method (Cassirer et al., 2017; Kamath et al., 2019). WADDL provided cycle threshold values corresponding to PCR diagnostic tests. Oropharyngeal swabs were gathered during each event except for the March 12th captive sampling, and were submitted to WADDL for bacterial culturing. Blood was collected by jugular venipuncture into PAXGene and serum separator tubes that were centrifuged within 4 h of collection (results presented in Bowen et al., 2022). Serum was separated and stored frozen in cryogenic vials until it was shipped frozen to WADDL. The sensitivity of the WADDL cELISA test is 90.7%, and its specificity is 95.8%.

At RGG, NMDGF personnel chemically immobilized 29 free‐ranging animals (19 female animals and 10 male animals) with BAM administered by a dart rifle between April 13th and May 13th, 2020. Blood and nasal swabs were collected, individuals were assigned a condition score based on palpation, coughing and nasal discharge status were recorded, and animals were fitted with GPS collars (Advanced Telemetry Systems, Isanti MN) for on‐going tracking. Samples were held frozen at NMDGF facilities and then shipped on ice to WADDL for diagnostic testing.

Body conditions at Hardware Ranch were estimated using ultrasound by measuring rump fat and loin thickness of the animals in similar locations as described for deer (Cook et al., 2007) and by palpation. Rump fat, which is the conventional target for ultrasound measurement of ungulate body condition (Cook et al., 2001, 2007; Stephenson et al., 1998), was very low in most animals at the first sampling event (median among positives = 1.25 mm; median among negatives = 1.00 mm). Therefore, we tracked loin thickness as a relative measure of condition changes within an animal in subsequent sampling events.

Five captive animals that died prior to euthanasia were necropsied by pathologists at the Utah Veterinary Diagnostic Laboratory in Logan, UT. The 13 remaining animals were euthanized on March 26th and necropsied by a veterinary pathologist‐researcher team.

#### 2.1.2 Epidemiological outcomes

We tracked survival of captive animals, and although no captive animal delivered a lamb prior euthanasia, we assessed pregnancy status throughout the disease event and weighed and examined fetuses postmortem to estimate fetal ages. Fetal age was estimated based on polished developmental benchmarks for domestic sheep (Sivachelvan et al., 1996) and published birthweights for Rocky Mountain bighorn lambs (Hogg et al., 1992).

The *M. ovipneumoniae* epidemic at RGG was allowed to play out in the absence of management intervention. Free‐ranging animals at RGG were observed by NMDGF biologists over 31 unique observation days between February 25th and July 18th, 2020. Epidemiological data included survival of the instrumented animals captured by NMDGF in April and May; survival of lambs born to instrumented ewes through mid‐summer; and aggregate lamb: ewe ratios in the summer and fall (gathered once in July and once in November). Field crews measured lamb: ewe ratios using protocols similar to those used in previous years, which allowed us to compare lamb: ewe ratios in 2020 to prior years.

#### 2.1.3 Observational scoring of clinical signs and reproductive status

Clinical signs were scored for the captive animals each day. Observers simultaneously watched all animals in the pen for 45 min and recorded signs including inappetance, nasal discharge, coughing (including number, quality, and pacing of coughs), and lethargy. Nasal discharge at Hardware Ranch was given a numeric score between 0 and 5. Shiny noses were given scores of 1, 2 indicated clear discharge from one nostril, 3 indicated clear discharge from both nostrils, 4 indicated purulent discharge from one nostril, and 5 indicated purulent discharge from both nostrils. Coughs were scored as 1 for isolated coughs, 2 for bouts of five or more consecutive coughs, and 2.5–4 for 2 or more bouts of 5 or more consecutive coughs, depending on depth of cough and number of bouts. Nose licking and head shaking were assigned scores of 1 if present and 2 if consistent throughout the observation period. Total daily scores for each animal were determined by summing the nasal, head shaking, and nose licking scores, and twice the individual's coughing score. Coughing scores were cumulative, with each coughing event contributing separately to the symptom score, so scores had no predefined upper‐bound. Individual daily symptom scores ranged from 0 to 17.5. Photograph records of noses (to track nasal discharge) and hips were gathered as frequently as possible to verify observer records. We did not visually score body condition because animals maintained winter coats throughout the study, but severe declines in body condition were noted on scoring sheets at the observer's discretion.

At RGG, observers recorded coughing (including quality) at the individual level, and nasal discharge status whenever viewers were close enough to see discharge clearly during the summer lamb surveys. However, no observer watched animals at both RGG and Hardware Ranch and scoring rubrics differed between the sites. Therefore, we only compare symptom trends, and not symptom burdens between the sites.

#### 2.1.4 Study termination and follow‐up

UDWR could not identify a suitable location to safely release the captive, infected animals, so the captive animals were euthanized on March 26th, 2020, 34 days after the initial diagnosis. Animals were immobilized with BAM as described above, and sampled live prior to euthanasia via gunshot to the head. Shots were positioned to limit damage to the sinuses, so heads could be assessed for gross evidence of sinus tumors (Fox et al., 2011) at the Utah Veterinary Diagnostic Laboratory. Animals were otherwise necropsied in full at Hardware Ranch by UDWR staff, accompanied by pathologists from UVDL and researchers from Utah State University. Antemortem sampling included collection of the full suite of samples described previously; postmortem sampling included bronchial junction swabs, extraction and measurements of the fetus, and full tissue collection, and gross assessment for sinus tumors.

### 2.2 Data analysis

#### 2.2.1 Dynamics of *M. ovipneumoniae* infection among the captive and free‐ranging animals

Although WADDL regards PCR results with cycle thresholds above 36 as “indeterminate,” we include raw values between 36 and 40 (*n* = 3) in this analysis. Our goal was to describe how pathogen load changed over the course of infection. As we relied on commercial diagnostic testing, we did not have standard curve values in hand to formally enumerate load at the individual level. However, we were able to track relative load among the samples using the 2^(−Ct') method (Livak & Schmittgen, 2001), using the minimum Ct recorded across all samples at both RGG and Hardware Ranch as the calibrator value. Once sample‐specific relative loads were calculated, we compared those relative loads among sampling events and age classes.

#### 2.2.2 Immune response to *M. ovipneumoniae*

Serological data analyses aimed to address three main objectives: (1) estimating time to seroconversion among the captive animals; (2) identifying sources of among‐individual variation in patterns of antibody signal strength; and (3) comparing serological patterns in the captive and wild settings.

The cELISA test for *M. ovipneumoniae* should not produce negative “percent inhibition” results, and negative results that are sometimes reported are artifacts of a formula applied to the laboratory‐generated data. Therefore, we first reset negative cELISA values to 0, and then added 1 to every reported value to allow for a log transformation. cELISA models were fit using the glm function in the stats package in R, with family set to “Gamma” and an identity link. We compared models with four covariate structures: days post‐detection, age, days post‐detection plus age, and days post‐detection plus linear and quadratic effects for age; assessed model assumptions through standard diagnostic plots; and critiqued model validity by examining predictions arising from each model. Models were compared using AIC, and coefficients were interpreted only for the most parsimonious model within the competitive model set (i.e., the set of models within 2 AIC points of the best‐performing model). Dispersion and shape parameter estimates were refined separately after model fitting using the gamma.shape function in R's MASS package (R Core Development Team, 2013), and those estimates were used in the Wald tests to assess coefficient significance.

We estimated the number of days until cELISA percent inhibition exceeded 40 (the WADDL cut‐off for classification as non‐negative) by fitting a Poisson regression model that treated days since February 22nd as a function of logged cELISA percent inhibition and individual age. We then used that model to predict the day upon which cELISA percent inhibition exceeded 40 across a spectrum of ages.

#### 2.2.3 Disease progression and severity of clinical signs

Symptom data analyses aimed to address three objectives: (1) estimating the lag between exposure and emergence of clinical signs among the captive animals; (2) describing the rise and fall of clinical signs in the captive setting; and (3) qualitatively comparing the timing of clinical signs between the two sites.

We estimated the time to emergence of clinical signs through a changepoint analysis that described symptom score as a piecewise‐linear function of days postexposure using the piecewise. Linear function in R's SiZeR package (Sonderegger, 2012). This analysis identified the most likely breakpoint in the pattern of clinical signs among the captive animals. We built bootstrapped confidence intervals to quantify uncertainty in the changepoint location and pre‐ and post‐changepoint slopes. We used this approach, as opposed to time until first recorded symptoms for each animal to account for occasional sneezes or nasal discharge that are expected among translocated and captive animals independent of *M. ovipneumoniae* infection.

We described the rise and fall of symptoms postonset by first subsetting the symptom data down to just those data arising after symptom onset (with onset identified using the changepoint identified above), and then fitting a linear model of symptom score as a function of linear and quadratic effects of days postexposure, along with a fixed effect for individual age and a random intercept for each individual.

We compared six different models of body condition (as measured by loin thickness) among the captive animals. All models assumed residual normality and included a random intercept for individual to account for repeated measurements of specific individuals. Fixed effect combinations included models with age; days post‐February 22nd; cELISA percent inhibition value; age, days post‐February 22nd and an age‐by‐days interaction term; cELISA, days‐post‐February 22nd and a cELISA‐by‐days interaction term; and a model with cELISA‐by‐days and age‐by‐days interaction terms. Models were fit using R's lme4 package (Bates et al., 2007).

## 3 RESULTS

### 3.1 Disease progression and epidemiological outcomes

All captive animals developed symptoms, although the timing and severity of symptoms varied (Figure 3a). Signs were initially mild, with coughing first observed 8 days postexposure. Once symptoms emerged, however, they intensified rapidly, and at the epidemic's peak, we observed over 19 coughing bouts (including one of 85 deep coughs) in a single affected individual—Eartag 35—over a single 45‐min observation period. Younger animals had lower clinical sign scores, and clinical signs emerged later in that group (contrast between purple and gold lines in Figure 3a).

Thirteen of the 15 animals that were studied in captivity at Hardware Ranch were censored prior to clearing infection through euthanasia on March 26th (Table 1). One of the individuals that died prior to March 26th (Eartag 30) jumped the fence during the capture event on March 12th and was euthanized on March 13th. A second (Eartag 28) died of pneumonia on March 25th. All of the captive ewes except for one 11‐month and one 23‐month ewe were pregnant (based on ultrasound at capture or post‐mortem examination), and most were in the third trimester of pregnancy throughout the disease event. Fetal age estimates varied, with the oldest lamb at nearly full term on March 26th, and the youngest estimated at approximately 55 days from parturition (ear vein was prominent, displayed hairs on eyelids, weight was approximately 20% of weight of a typical full‐term bighorn ram lamb). None of the captive females lambed while in captivity, so we have very few ultimate epidemiological outcomes. Removing the nonpneumonia mortalities yielded a per‐animal mortality probability of .071 in captivity (95% binomial confidence interval = [0.01, 0.34]).

**TABLE 1:** Longitudinal data from 15 captive female bighorn sheep. “Tag” is eartag number. “Age” is estimated from tooth eruption and wear patterns. “Ct” is the WADDL‐derived cycle threshold from a real‐time PCR for *M. ovipneumoniae* (40 corresponds to no detection). “%I” is percent inhibition from the WADDL cELISA serological test (values >40 are regarded as indeterminate, and >50 are regarded as seropositive). “Rump fat” is an ultrasound‐based rump fat measurement in mm. “Loin” is an ultrasound‐derived measure of loin thickness in mm. “Weight” is weight in kilograms

Tag	Age	Feb 22nd						March 12th				March 27th				
		Ct	%I	BCS	Rump fat	Loin	Weight	Ct	%I	BCS	Loin	Ct	%I	BCS	Loin	Weight
26	6.5	40	−0.73	2.5	1	35	70	24.42	0.04	2	31	27.7	76.1	1.75	29	66
27	6	40	−6.31	2	0	38	65	28.18	−4.55	2	28	28.7	40.5	1.5	27	51
28	7.5	40	4.86	2.25	0	37	56	25.76	6.90	1.75	26	Mortality—pneumonia, 03/25/20				
30	4+	40	−35.85	NA	2	38	73	38.11	Mortality—jumped fence during second capture, then euthanized							
31	2.5	40	3.56	2.5	2	36	58	29.47	23.02	2	31	30.5	18.2	1.75	32	57
35	5.5	40	9.58	2.5	2	42	78	33.07	46.06	2.25	31	33.3	60.8	2.5	32	72
36	6	40	19.45	2	1	38	70	32.48	−4.68	1.75	29	29.1	21.6	1.5	27	62
38	7.5	40	13.75	2	1	37	65	33.1	6.90	2.25	32	28.4	39.8	2	30	62
39	4	40	22.42	2	2	38	60	35.11	−7.48	2	32	28.3	−1.4	2.25	31	57
40	0.9	40	−7.70	2.5	0	34	48	30.65	15.79	2	31	40	13.1	2	30	40
41	6	40	−8.59	2.25	1	38	68	23.96	15.96	1.75	26	31.9	67.4	1	24	60
42	3.5	40	14.42	2	1	35	55	28.6	2.91	2.5	34	30.8	11	1.75	28	50
43	1.5	40	−10.16	2.75	1	35	55	26.69	39.40	2.5	35	28.1	54.2	2.25	32	51
45	4+	40	−3.82	3.25	2	42	75	35.69	25.95	2.5	35	40	84.8	2	34	69
46	2.5	40	−5.31	2.5	1	36	55	26.17	29.68	2.25	33	34.1	60.8	1.75	28	47

Ewes in the free‐ranging population gave birth throughout March, April, and early May. Twenty‐three of the 29 radiocollared animals remained alive as of March 2nd, 2021, and five of the six mortalities were due to hunter harvest. No additional adult pneumonia mortalities were identified within the herd. However, the RGG lamb:ewe ratio on November 12th, 2020 was 22:100, as compared to 48:100 in 2019, and an average of 53:100 from 2007–2019. Within 2020, the lamb:ewe ratio decreased between two similarly conducted ground surveys in July (29:100) versus November (22:100). Parturition rates were high. Lambs were confirmed for 16 of the 19 marked ewes in the months before the July survey, and the three ewes with unknown lamb status probably gave birth to lambs that went undetected. Lamb:ewe ratios remained low in the December 2021 survey (21 months after the first detection of *M. ovipneumoniae* in the herd), at 22 lambs:100 ewes. In aggregate, this event imposed very little mortality burden on free‐ranging adults (95% binomial confidence interval for disease‐induced adult mortality = [0.00, 0.21], which overlaps with the confidence interval from the captive animals prior to euthanasia). However, lamb survival declined to the range of postdie‐off lamb:ewe ratios reported elsewhere (Cassirer et al., 2018). Observers reported symptoms including coughing in lambs on several occasions during summer field observations.

### 3.2 Dynamics of *M. ovipneumoniae* infection in the captive and free‐ranging herds

Multilocus strain typing (Cassirer et al., 2017; Manlove et al., 2019) of *M. ovipneumoniae* sequences from six different animals indicated that the *M. ovipneumoniae* strain belonged to a clade associated with domestic goats (Kamath et al., 2019). We assumed that the captive animals were exposed on February 22nd during transit from RGG to Utah because there was no prior diagnostic evidence of infection in the RGG herd, and because the timelag until symptom emergence was consistent with *M. ovipneumoniae* incubation periods reported elsewhere (Besser et al., 2014). At the sampling event on March 12th, all animals were PCR‐positive for *M. ovipneumoniae*, except for one individual who was indeterminate (Eartag 30, the animal that escaped). We tracked temporal dynamics of pathogen load within animals by comparing measured load to the maximum load (minimum cycle threshold, “Ct”) detected across the entire study. The minimum Ct was 23.1, obtained from a bronchial junction swab from Eartag 27 at Hardware Ranch; the minimum Ct from a nasal swab was 23.96, from Eartag 41 at Hardware Ranch on March 12th. Samples were calibrated against the minimum bronchial junction value.

**FIGURE 1:**
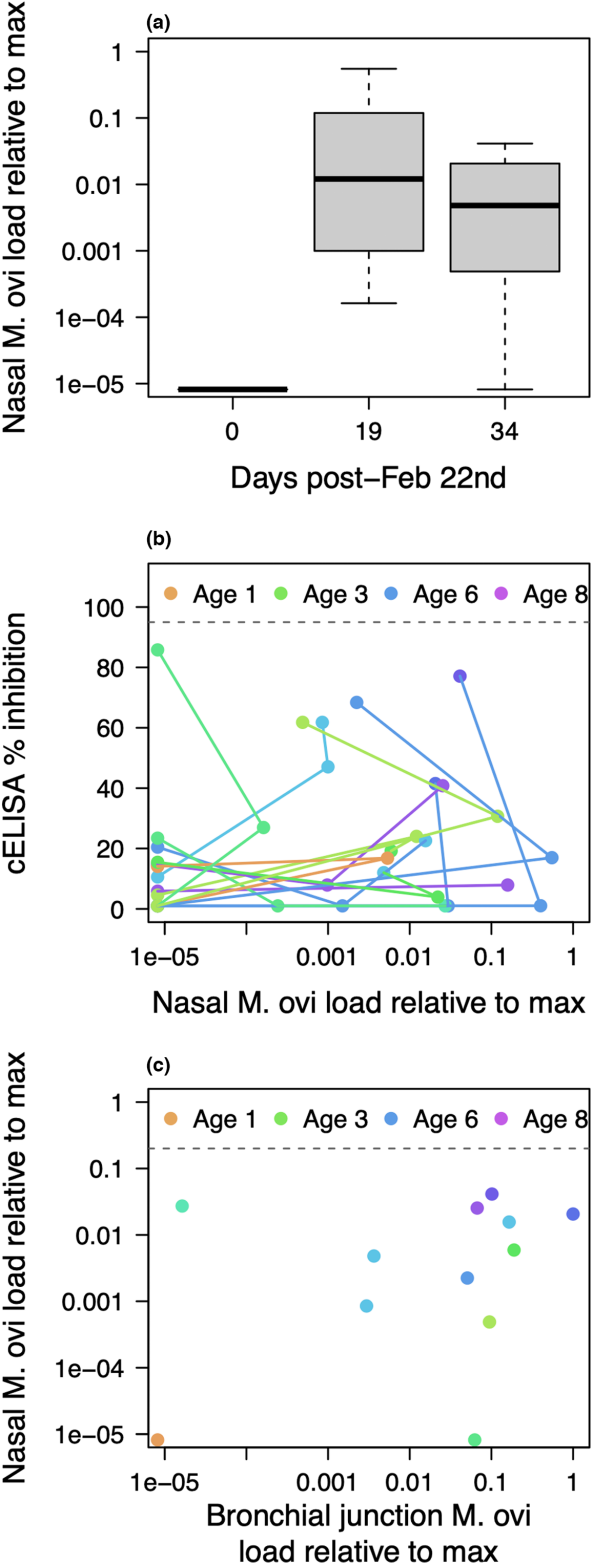
Dynamics of *M. ovipneumoniae* (“M. ovi”) infection in the captive bighorns over 3 sampling events between February 22 and March 27, 2020. (a) *M. ovipneumoniae* load relative to the maximum load observed in this study obtained from nasal swabs associated with captive animals over each sampling event. A relative load of 1e‐05 approximately corresponds to a PCR‐negative animal with a cycle threshold (Ct) of 40. All captive animals were PCR‐negative on the first sampling event. (b) Relative *M. ovipneumoniae* loads and serological values for each individual at each sampling event at hardware ranch. Points within an individual are connected. All individuals started uninfected (left‐hand side of the *x*‐axis), with relatively low cELISA values (points low on the *y*‐axis) in the first sampling event. As infections progressed, first relative loads and then antibody signal strengths increased, shifting points to the right on the *x*‐axis, and then up on the *y*‐axis. Animals that completely cleared infection returned to a relative load of approximately 1e‐05, but with higher cELISA values during the third sampling event. (c) Relative *M. ovipneumoniae* loads in the nasal passage and bronchial junction in the final sampling event

*M. ovipneumoniae* load had declined in most captive animals by the destructive sampling event 34 days post‐exposure (corresponding to higher *M. ovipenumoniae* Ct values; Figure 1a), although load rose incrementally in three individuals (Eartags 36, 38, and 39). Older animals had slightly higher pathogen burdens in their noses during infection than younger animals (blue lines rise to higher values on the *y*‐axis in Figure 1b than green lines), although the correlation between age and maximum load did not differ substantially from 0 (Pearson's *r* = 0.22; 95% CI = [−0.38, 0.69]). *Pasteurella multocida* was cultured from oropharyngeal swabs (detected in 10 of 10 sampled individuals) and lung tissue (detected in 10 of 13 sampled individuals) at necropsy. No gross evidence of sinus tumors was detected in any animals upon necropsy.

Upper and lower respiratory tract infection status (measured through Ct values from nasal and bronchial junction swabs shown in Figure 1c) generally agreed within an animal, although this agreement was not absolute. Two animals had discrepant outcomes. One (Eartag 45) had very low levels of *M. ovipneumoniae* at the bronchial junction, but was positive on the nasal swab; another (Eartag 39) continued to harbor *M. ovipneumoniae* in the bronchial junction, but had no detectable nasal infection. A third animal (Eartag 40, the youngest animal in the captive group) apparently cleared infection from both the nose and the bronchial junction by the final sampling event. Eartag 40's cELISA percent inhibition values also never approached “detectable” levels (cELISA %I the final sampling was 13.1).

A total of 21 of the RGG animals were PCR‐positive for *M. ovipneumoniae* at capture, and another three were PCR‐negative but showed serological signals consistent with exposure (cELISA percent inhibitions of 69, 64, and 64). All three were under four years of age (1, 3, and 3.5), although a formal statistical relationship between age and probability of clearance could not be detected at this sample size. However, the difference in age was detectable in an analysis that pooled the captive and free‐ranging animals (*p* = .048 in a randomization test of animal ages). Among the animals that cleared infection, one was male and three were female.

### 3.3 Immune response to *M. ovipneumoniae*

**FIGURE 2:**
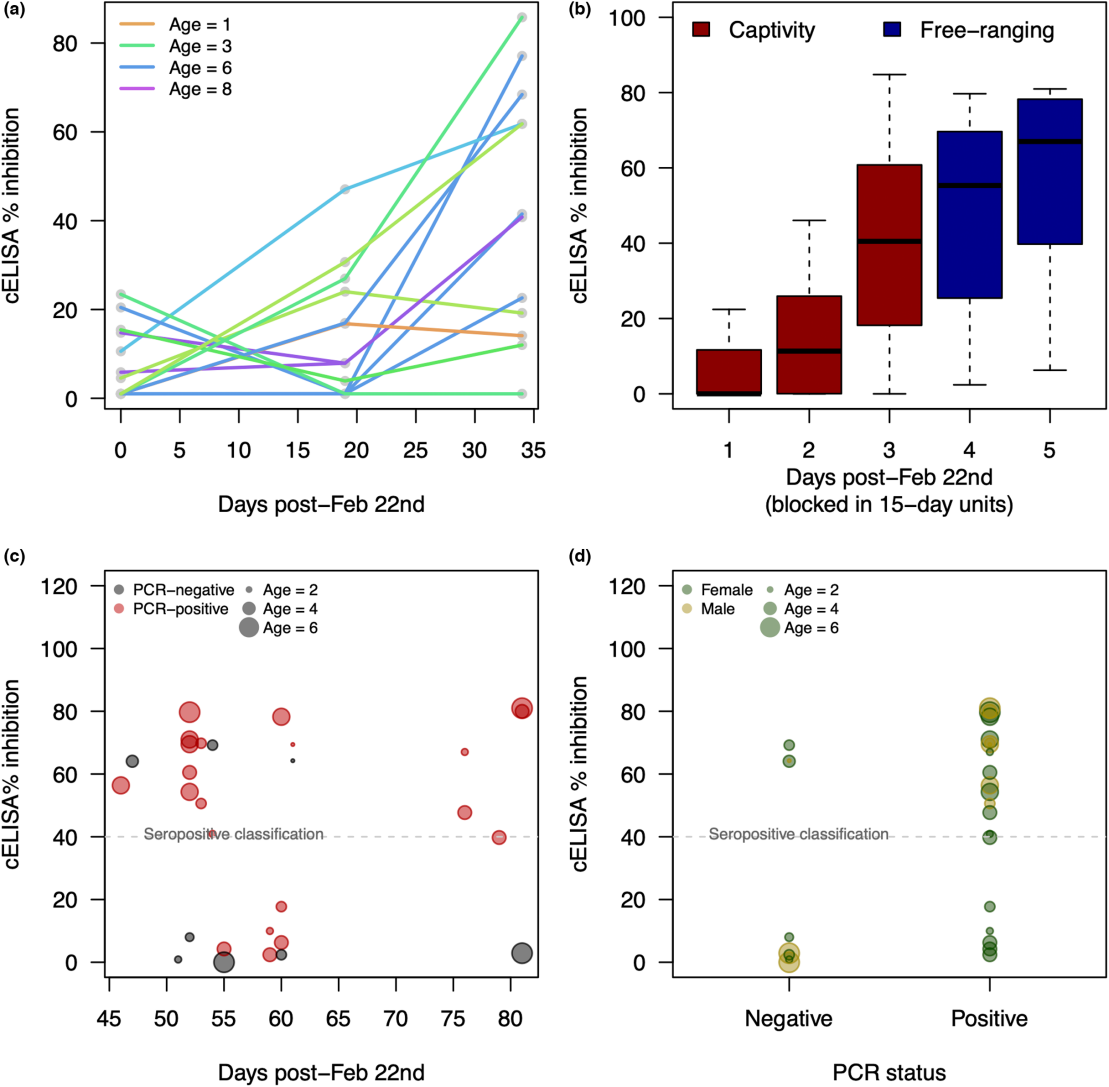
Serological dynamics of *M. ovipneumoniae* infection. (a) *M. ovipneumoniae* cELISA percent inhibition through time by animal among the captive individuals at Hardware Ranch. (b) Aggregate *M. ovipneumoniae* cELISA percent inhibition values through time among PCR‐positive animals across both sites. (c) *M. ovipneumoniae* cELISA percent inhibition as a function of date of sampling for the RGG animals. Point colors indicate whether the animal was also PCR‐positive for *M. ovipneumoniae* (red), or PCR‐negative (gray). (d) *M. ovipneumoniae* cELISA percent inhibition by PCR status among the animals captured at RGG. The dashed lines in panels c and d indicate the 40% inhibition level that WADDL uses as a cut‐off value for classifying animals as seropositive

Patterns of increasing antibody signal varied weakly with age among the captive females at Hardware Ranch (Figures 1b and 2a), with seven animals failing to seroconvert by the time of euthanasia (all lines ending at a *y*‐coordinate below 40 in Figure 2a). AIC‐based comparisons showed no substantial model improvements when age was included as a predictor (Table 2). Predictions from the Poisson regression model of days since February 22nd as a function of cELISA percent inhibition and age suggested that the typical female would exceed 40% inhibition on the cELISA test at 25 days postexposure (95% CI for the average days to crossing = [23, 27]; Table S1).

**TABLE 2:** Comparison of plausible models for cELISA signal strengths

Model	AIC	Δ AIC	AIC weight
Age	309.62	12.36	0.00
Days post‐exposure	297.26	0.00	0.47
Days post‐exposure + Age	298.16	0.90	0.30
Days post‐exposure + Age + Age^2^	298.73	1.47	0.23

Antibody expression appeared to follow relatively similar time courses in the captive and free‐ranging settings. Although sampling was not concurrent across the two sites, general trends in antibody expression among animals with active *M. ovipneumoniae* infections aligned well (e.g., Figure 2b). In the free‐ranging RGG herd, 11 of 15 animals sampled prior to April 20th had already seroconverted to a cELISA percent‐inhibition value above 40 prior to sampling (Figure 2c). The disease event was still underway, however, as evidenced by the five PCR‐positive animals that had yet to seroconvert (Figure 2c,d). Three RGG animals had antibodies to *M. ovipneumoniae* but no evidence of current infection, suggesting they may have already cleared infection (Figure 2d).

We did not have enough data to determine whether immune responses varied between the sexes at RGG. PCR‐positive males had marginally higher antibody levels at capture than PCR‐positive females (median percent inhibition among PCR‐positive males = 69.5, IQR = [62.9, 74.9]; among PCR‐positive females = 44.3, IQR = [11.9, 65.4]). This difference could be due to random variation in when individuals were sampled relative to their exposure events, which were not well‐known at RGG.

### 3.4 Severity of clinical signs

The changepoint analysis suggested a significant shift in symptoms among the captive animals at 12.9 days postexposure (95% bootstrapped CI [11.50, 18.00]). Symptom scores did not increase significantly with time prior to that day (*β*_days post‐exposure pre‐changepoint_ = 0.04; 95% CI [−0.01, 0.25]), but increased after it (*β*_days post‐exposure post‐changepoint_ = 0.34; 95% CI [0.33, 0.50]).

Symptom scores post‐onset (i.e., 12.9 days postexposure and beyond) were best described by a model with linear and quadratic effects of time, and an additive effect of individual age. The quadratic effect was significantly negative, suggesting that symptoms would have peaked at about 36 days postexposure (*β*_days^2_ = −0.02, SE = 0.01). Symptom scores were generally higher among older animals (*β*_additional year of age_ = 0.22, SE = 0.19), although this effect was only marginally significant, and the timing and rate of symptom increase through time did not vary significantly according to age.

**FIGURE 3:**
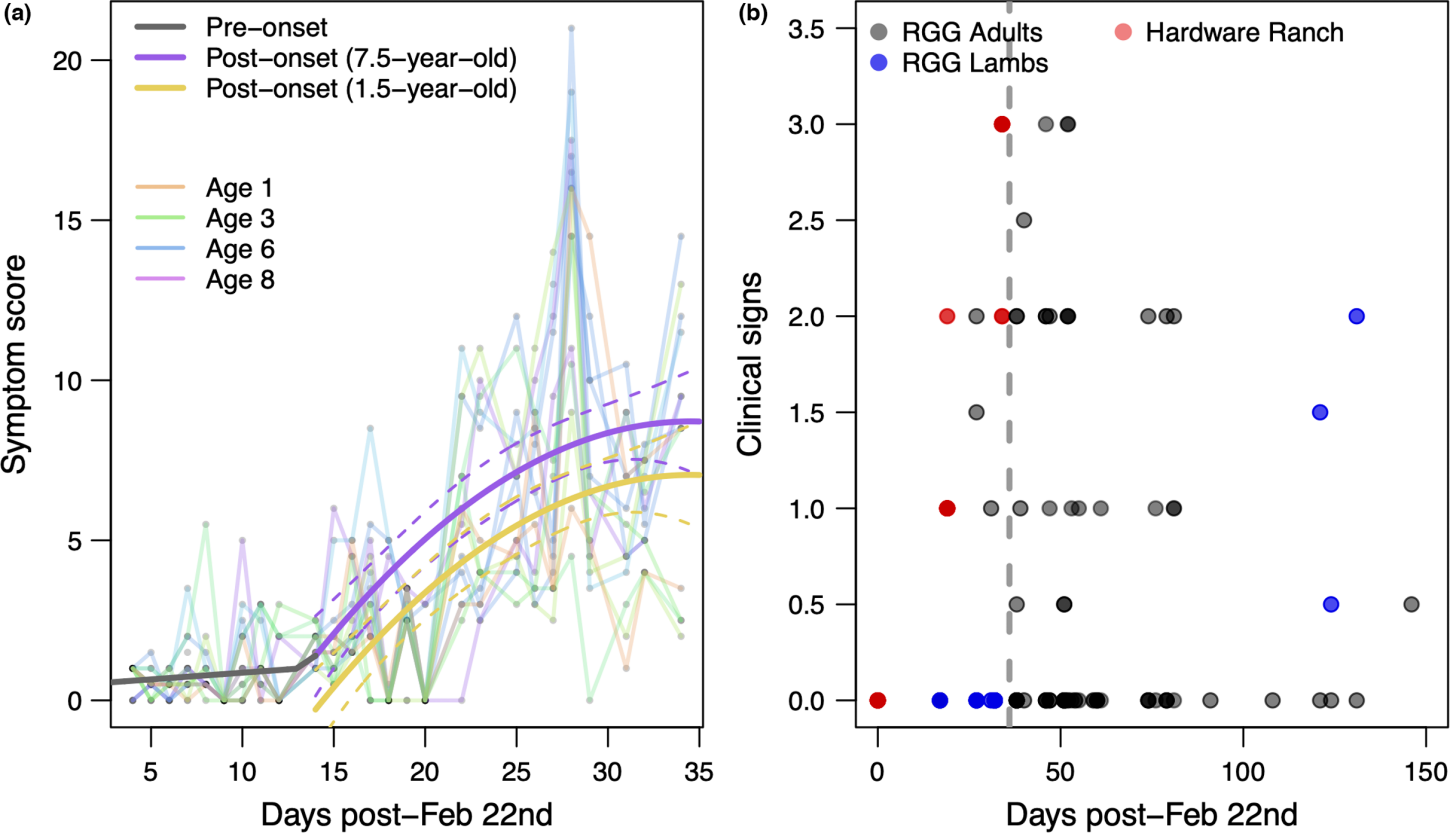
Clinical signs progression. (a) Clinical signs among the captive animals at hardware ranch. Transparent lines show observed scores for each animal. The bold gray line shows presymptom‐onset patterns. Bold purple and gold lines show expected symptom scores for 7.5‐year‐old and 1.5‐year‐old animals, respectively, with dashed lines indicating 95% confidence intervals for each fit. (b) Clinical signs (scores are recalibrated to compare data from RGG and hardware ranch; a score of 3 was assigned to animals that were coughing severely at capture, a score of 0 was assigned to animals with no clinical signs). The increase in clinical signs over summer in lamb groups is consistent with endemic‐phase *M. ovipneumoniae* dynamics reported elsewhere (Cassirer et al., 2017). The gray vertical line in B is at 36 days post‐February 22nd, the estimated lag until peak symptoms from the captive data

Symptoms were scored slightly differently at RGG and Hardware Ranch, thus, we did not compare specific symptom scores across sites. Among the RGG animals, clinical signs declined steadily from April 13th to May 13th. However, clinical signs were observed sporadically (in 7 of 73 groups observed from May 1st to July 18th) during the summer of 2020 in both ewes and lambs (Figure 3b).

**FIGURE 4:**
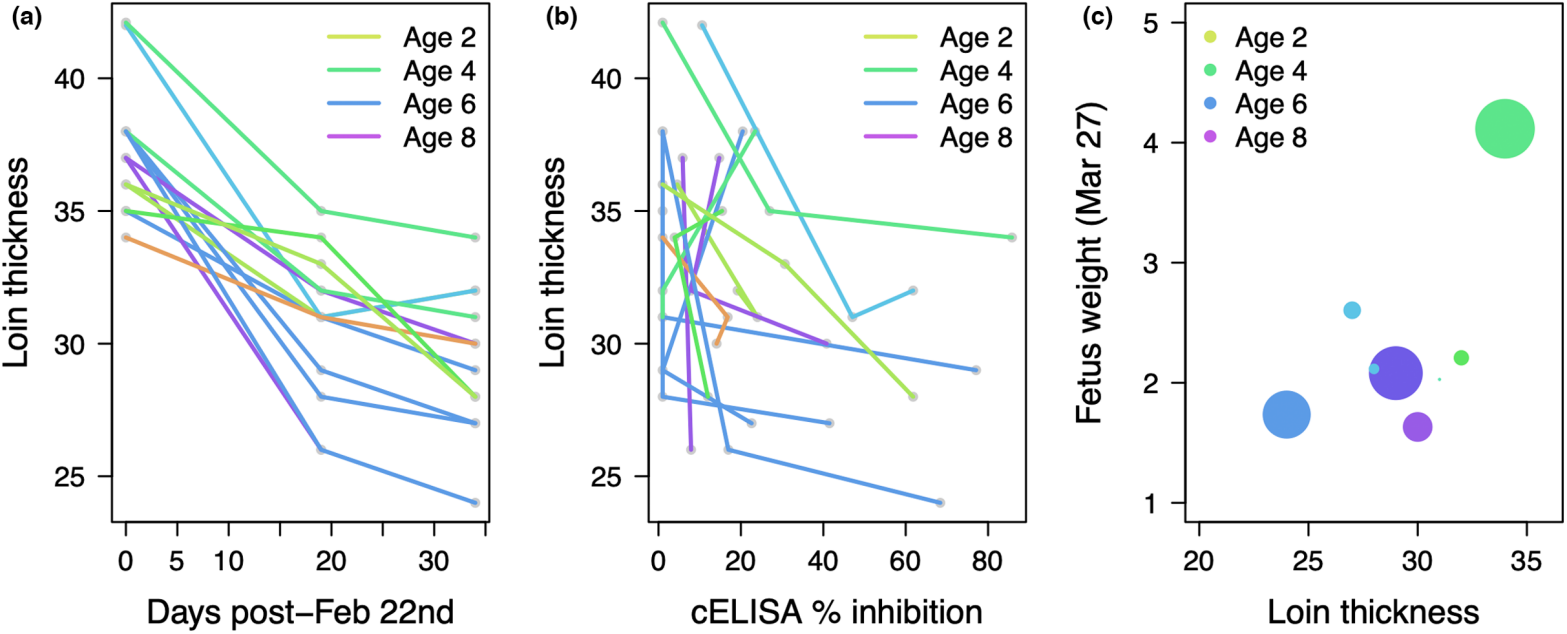
Patterns of loin thickness and *M. ovipneumoniae* serology among the captive animals. (a) Loin thickness through time by animal. (b) Loin thickness by *M. ovipneumoniae* cELISA percent inhibition by animal. (c) Fetus weight at necropsy as a function of maternal loin thickness at the final sampling event (point sizes correspond to cELISA percent inhibition at that same event; larger circles indicate % inhibition of ~80; smallest circles indicate % inhibition of ~20)

The six captured animals that were PCR‐ or cELISA‐positive for *M. ovipneumoniae* exposure on the February 22nd capture event showed similar, and usually more, fat reserves than captured animals that were PCR‐ and cELISA‐negative for *M. ovipneumoniae* on the first capture (median loin thickness among positives = 37.0 mm; median loin thickness among negatives = 37.5 mm; Figure S1). Loin thickness was correlated with rump fat and aggregate body weight (*r* = 0.52, 95% CI = [−0.04, 0.83] between loin thickness and rump fat; *r* = 0.53, 95% = CI [0.18, 0.77] between loin thickness and body weight; Figure S2), and was correlated within animals through time (Figure 4a)

Upon entering captivity, loin thickness declined significantly with days in captivity, although this effect diminished through time. Declines were more severe among older animals than younger animals (Figure 4a), and patterns in loin thickness were better explained by age than serology (Figure 4a vs. 4b). The best model of loin thickness included age, days post‐exposure, and an age x days‐post‐exposure interaction (AIC = 123.40). The next‐best model included days post‐exposure only (AIC = 127.13). Loin thickness in the final sampling event was positively correlated with fetus weight at necropsy (Figure 4c), although this relationship was estimated with substantial uncertainty (*r* = 0.58; 95% CI = [−0.21, 0.91]). No parallel decline in body condition was documented at RGG.

## 4 DISCUSSION

This study describes in‐host dynamics of a novel *M. ovipneumoniae* introduction into a Rocky Mountain bighorn sheep population, and compares epidemic outcomes in two different ecological contexts. We estimated a 12.9‐day incubation period of very low clinical signs, followed by a period of symptom expansion that was estimated to peak around 36 days postexposure. Seroconversion occurred an average of 24.9 days postexposure among the females held in captivity. Symptom, pathogen load, and serological dynamics varied among individuals, and in one animal, the infection was insufficient to generate an immune response classically indicative of exposure. Younger animals were more likely to clear the pathogen prior to sampling than older animals. Gross patterns of antibody signal strength and symptom emergence were consistent across the two very different environments, suggesting that pathogen and host‐specific factors were important determinants of *M. ovipneumoniae* epidemiology, at least in this case.

### 4.1 Comparing disease outcomes in captive and wild settings

The epidemic progressed in very different environments at RGG and Hardware Ranch. While the RGG animals were free‐ranging, the captive animals experienced unnaturally high contact rates, along with stress from translocation, living in captivity, dietary shifts, etc. Moreover, data collection at RGG and Hardware Ranch were not perfectly aligned in time, and may reflect different phases of the disease event. While discrepancies in timing may explain some of the differences between the sites, they cannot explain the difference in mortality burdens (18% in captivity vs. 0% in the free‐ranging herd). Despite the contextual differences, disease dynamics at the two sites had several similarities. First, antibody signal strengths at Hardware Ranch and RGG followed relatively similar patterns (Figure 2b). The captive data are our only line of information surrounding the period of increasing antibody signal strength during the disease event (Figure 2a); the RGG data, which arose slightly later in the epidemic, generally showed relatively strong antibody signal strength, especially in the later samples. When temporally aligned with one another, however, the patterns at the two sites are relatively seamless (Figure 2b). The timing at which clinical signs peaked was also consistent between the two sites: the plateau in clinical signs late in the captive study (Figure 3a) is consistent with subsequent declining signs in the RGG animals after about April 1st (Figure 3b). The estimated date of maximum symptom burden based on the Hardware data is consistent with the observed maximum symptom burden date at RGG (Figure 3b). However, comparing the intensity of clinical signs is not possible using these data as observation opportunities and overall epidemic intensities may have differed between the captive and wild settings.

### 4.2 Epidemiology and individual heterogeneity in response to infection

These data provide a unique view of a “goat clade” *M. ovipneumoniae* strain invading a bighorn herd. Strains derived from domestic goats (as opposed to domestic sheep) cluster genetically (Kamath et al., 2019; Maksimović et al., 2017), and have been shown to produce less‐severe infections in experimental settings (Besser et al., 2017). In the free‐ranging RGG animals, the strain studied here posed a low mortality burden on adult animals relative to what has been reported elsewhere for strains from the “goat clade” (Cassirer et al., 2017), although disease burden on lambs was in line with burdens from more severe *M. ovipneumoniae* strains (Cassirer et al., 2018; Manlove et al., 2016). Thus, this report suggests a potentially lower—but non‐negligible—burden of “goat clade” *M. ovipneumoniae* on bighorn sheep.

Importantly, several animals studied here failed to seroconvert, even after PCR‐confirmed infection with *M. ovipneumoniae* (a pattern consistent with Johnson et al., 2022). This underscores the variation in individual immune responses to this pathogen. The processes leading to this variation merit further investigation.

Body weight and loin thickness declined during infection among the captive animals (similar to patterns reported in Besser et al., 2021). We cannot determine whether those declines were due to environment or disease in this study. However, loin thickness in the animals that were already infected during the first capture event did not differ significantly (and were typically in incrementally better condition, Figure S2) from loin thickness of uninfected animals during that same sampling event, perhaps providing a weak indication that *M. ovipneumoniae* alone was not responsible for the body condition declines. Although loin thickness declined with age, younger animals started with lower loin thicknesses, and there may have been a limit in how much loin could be lost.

While all individuals in the captive setting developed infections, symptom severity, infection duration, and antibody responses varied (even within a single host sex, from a single host source population). One proximal driver of this variation was age: younger animals mounted faster antibody responses, had less‐severe declines in body condition, and experienced slightly lower pathogen loads. However, the ultimate processes generating differences among ages are not clear.

Other studies of bighorn sheep pneumonia have reported associations between age and chronic carriage rates (Plowright et al., 2017), and age and transmission (Manlove et al., 2017), though the second could not separate behavioral drivers (e.g., young animals had lower contact rates) from in‐host factors (e.g., young animals bore lower pathogen burdens or secreted lower volumes of pathogen into the environment). The data presented here suggest that immunodynamics vary with age in the bighorn sheep‐*M. ovipneumoniae* system, consistent with patterns observed in other free‐ranging sheep populations (Nussey et al., 2012; Watson et al., 2016).

In the wild setting, we saw differences among sexes in pathogen load and antibody response (both were higher in males). However, our data cannot determine whether those effects are due to differences in date of infection or to differences in immune function between the sexes.

### 4.3 Study limitations

The data analyzed here arose through a natural experiment, and several factors limit our ability to directly compare individual animals or sites. First, we speculate that most captive animals were exposed on or very shortly after the first capture event on February 22nd, but infection timing is not definitively known. Some animals may have avoided initial exposure, but avoidance would have probably been random (young and old animals were well‐mixed in the trailer), and should not substantially confound our analyses.

Timing of exposure in the free‐ranging RGG herd is less certain than among the captive animals, thus the symptom dynamics in Figure 3b represent individuals at varying stages of infection. Nevertheless, the clear decline in symptoms at the RGG herd suggests that most animals resolved acute symptoms of infection within no more than 100 days of exposure. This is fairly consistent with the symptom peak ~36 days postexposure in the captive animals.

Finally, most of data assessed here are from females of reproductive age, and the longitudinal patterns we describe should not be extended to males without validation. A few lines of evidence suggest that symptom dynamics in males may correspond closely to those of females (e.g., the 10 males captured at RGG were not outliers in symptom expression), but we lack sufficient replication to fully document those differences here.

### 4.4 Implications for management

This natural experiment provided a few observations that are relevant for management. The six translocated animals that tested positive for *M. ovipneumoniae* on the first sampling event showed no clinical signs of disease. This underscores the importance of laboratory‐based testing, as opposed to gross field observations, in determining infection status prior to translocation. Overnight testing prior to release proved informative here, and may be a general best‐practice to limit accidental introduction of *M. ovipneumoniae*. Moreover, parsimony suggests that those apparently asymptomatic animals were able to transmit *M. ovipneumoniae* to otherwise uninfected animals during the translocation event. Granted, the stress and proximity of the translocation deviated from “natural” transmission conditions, but at face value, this observation suggests that culls targeting animals with obvious symptoms may not always eliminate the pathogen. Follow‐up timing and measurable ultimate outcomes differed between the two settings studied here, but the data that could be aligned suggest fairly consistent disease dynamics under different environmental (and probably stress) conditions. This provides a gentle indication that host‐ or pathogen‐specific factors may be more important determinants of disease severity in this system than factors tied to environmental context.

Longitudinal data on in‐host measurements hold huge value for this system—and wildlife disease management more generally—as they provide context on the repeatability of serological and PCR values over time. Our data suggest that researchers wishing to document early infections and immune response during an emerging bighorn sheep disease event should concentrate sampling efforts during the event's first month. If the intent is to measure epidemic size, it might be better to wait several months until most animals have had an opportunity to seroconvert and potentially clear infection. In this study, very few animals cleared the pathogen prior to sampling. Managers planning test‐and‐remove efforts surrounding new disease events should proceed with caution within the epidemic's first few months to avoid removing animals who might eventually recover (especially if the disease‐induced mortality burden of the event among adults is low). Lastly, data on incubation period, timing of seroconversion, and timing of clinical signs have direct utility for epidemiological models that help guide management (e.g., Almberg et al., 2021). We hope these data can aid research and management going forward.

## AUTHOR CONTRIBUTIONS

**Kezia Manlove:** Conceptualization (lead); data curation (equal); formal analysis (lead); investigation (equal); methodology (lead); project administration (lead); resources (supporting); software (lead); supervision (equal); validation (lead); visualization (lead); writing – original draft (lead). **Annette Roug:** Conceptualization (supporting); data curation (supporting); funding acquisition (supporting); investigation (equal); project administration (supporting); resources (supporting); writing – review and editing (supporting). **Kylie Sinclair:** Conceptualization (supporting); data curation (supporting); investigation (supporting); methodology (supporting); writing – review and editing (supporting). **Lauren E Ricci:** Conceptualization (supporting); data curation (supporting); methodology (supporting); project administration (supporting); writing – review and editing (supporting). **Kent R Hersey:** Conceptualization (supporting); funding acquisition (supporting); investigation (equal); methodology (supporting); project administration (supporting); resources (supporting); supervision (supporting); writing – review and editing (supporting). **Cameron Martinez:** Investigation (supporting); project administration (supporting); writing – review and editing (supporting). **Michael Martinez:** Data curation (supporting); investigation (supporting); project administration (supporting); writing – review and editing (supporting). **Kerry Mower:** Conceptualization (supporting); data curation (supporting); funding acquisition (supporting); investigation (supporting); project administration (supporting); writing – review and editing (supporting). **Talisa Ortega:** Conceptualization (supporting); data curation (supporting); investigation (supporting); project administration (supporting); writing – review and editing (supporting). **Eric Rominger:** Conceptualization (supporting); funding acquisition (supporting); investigation (equal); project administration (supporting); writing – review and editing (supporting). **Caitlin Ruhl:** Conceptualization (supporting); funding acquisition (supporting); investigation (supporting); project administration (supporting); writing – review and editing (supporting). **Nicole Tatman:** Conceptualization (supporting); funding acquisition (equal); investigation (supporting); project administration (supporting); writing – review and editing (supporting). **Jace Taylor:** Conceptualization (equal); data curation (supporting); funding acquisition (supporting); investigation (supporting); project administration (equal); resources (supporting); writing – review and editing (supporting).

## FUNDING INFORMATION

Funding for this project was provided by Utah division of wildlife resources, new Mexico Department of Game and Fish, and Utah State University faculty start‐up support for KRM.

## CONFLICT OF INTEREST

None declared.

## Supporting information


**TABLE S1** Model coefficient estimates for the days‐to‐seroconversion model.
**FIGURE S1.** Loin thickness, rump fat, and weight at original capture for animals that tested PCR‐ or Seropositive for *M. ovipneumoniae* on the February capture event (group “Pos”) to animals who did not test positive on that event (“Neg”).
**FIGURE S2.** Relationship between rump fat and loin thickness (A) and body weight and loin thickness (B). Rump fat was only measured during the first sampling event and was excluded in subsequent samplings because it did not vary. Weight was only measured on the first and last sampling because of timing constraints in the middle sampling event.

## Data Availability

The data that support the findings of this study are openly available through Dryad at 10.5061/dryad.0vt4b8h1p.
