# Supplementary material for: Bighorn sheep show similar in‐host responses to the same pathogen strain in two contrasting environments
Source: Ecol Evol. 2022 Jul 17;12(7):e9109. doi: 10.1002/ece3.9109 (PMC9288933; doi:10.1002/ece3.9109)
Supplement: Supplementary file 1 — TABLE S1 Model coefficient estimates for the days‐to‐seroconversion model. FIGURE S1. Loin thickness, rump fat, and weight at original capture for animals that tested PCR‐ or Seropositive for M. ovipneumoniae on the February capture event (group “Pos”) to animals who did not test positive on that event (“Neg”). FIGURE S2. Relationship between rump fat and loin thickness (A) and body weight and loin thickness (B). Rump fat was only measured during the first sampling event and was excluded in subsequent samplings because it did not vary. Weight was only measured on the first and last sampling because of timing constraints in the middle sampling event. [file ECE3-12-e9109-s001.docx]

## Appendix

### Model of days to seroconversion

The model was of the following form:

log(Days post-exposure_i_ ) = β_0_ + β_1_ * ln(cELISA % inhibition_i_ ) + ε_i_ . Model coefficient estimates are shown in Table S1. The model was fit only to data from the captive females at Hardware Ranch. Age was not predictive in this context, and we generate predictions for four-year-old animals only in the main text.

|  | Estimate | Standard error | Z-value | p-value from Wald’s test |
| --- | --- | --- | --- | --- |
| Intercept | 1.89 | 0.346 | 5.52 | <0.0001 |
| ln(cELISA % inhibition) | 0.36 | 0.11 | 3.26 | 0.002 |

**Table S1.** Model coefficient estimates for the days-to-seroconversion model.

### Starting body condition


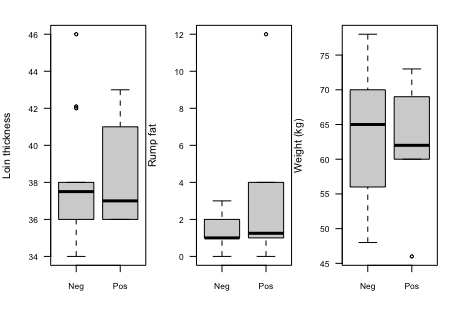


**Figure S1.** Loin thickness, rump fat, and weight at original capture for animals that tested PCR- or Seropositive for *M. ovipneumoniae* on the February capture event (group “Pos”) to animals who did not test positive on that event (“Neg”).


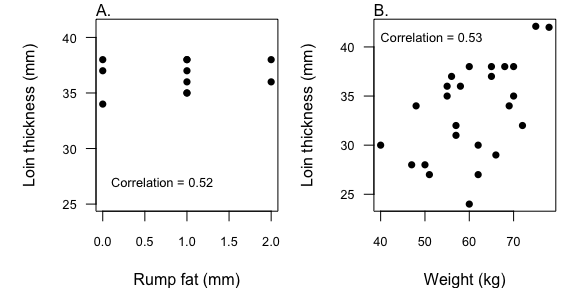


**Figure S2.** Relationship between rump fat and loin thickness (A) and body weight and loin thickness (B). Rump fat was only measured during the first sampling event, and was excluded in subsequent samplings because it did not vary. Weight was only measured on the first and last sampling because of timing constraints in the middle sampling event.
